# Surprise Egg in the Nasopharynx: Basal Cell Adenocarcinoma

**DOI:** 10.7759/cureus.23661

**Published:** 2022-03-30

**Authors:** Yusuf Ç Kumbul, Erdoğan Okur, Vural Akın, Veysel A Ayyıldız, Mehmet Kıran

**Affiliations:** 1 Department of Otorhinolaryngology and Head and Neck Surgery, Suleyman Demirel University, Faculty of Medicine, Isparta, TUR; 2 Department of Radiology, Suleyman Demirel University, Faculty of Medicine, Isparta, TUR; 3 Department of Pathology, Suleyman Demirel University, Faculty of Medicine, Isparta, TUR

**Keywords:** salivary gland malignancy, adenoma, minor salivary glands, basal cell adenocarcinoma, nasopharynx

## Abstract

Basal cell adenocarcinoma is a rare salivary gland neoplasm. It is most commonly seen in the parotid gland, and its involvement in the minor salivary glands or upper respiratory tract is very rare. Surgical excision and/or radiotherapy are the mainstay treatment modalities. The nasopharynx is an unusual location for salivary gland basal cell adenocarcinoma. In this case report, the nasopharyngeal punch biopsy of a 60-year-old male patient was reported as salivary gland adenoma, but the final pathological diagnosis was changed to basal cell adenocarcinoma after endoscopic nasopharyngectomy. The clinical, radiological, and histopathological features of the case are presented in light of the literature. In addition, diagnostic clues are emphasized in cases of suspected non-epithelial tumors of the nasopharynx.

## Introduction

The main headings in the World Health Organization's (WHO) classification of nasopharyngeal tumors are carcinomas, salivary gland tumors, benign and borderline lesions, soft tissue tumors, hematolymphoid tumors, and notochordal tumors. Among these headings, basal cell adenocarcinoma (BCAC) is included in salivary gland tumors [1]. BCAC synonyms are basal cell adenocarcinoma ex monomorphic adenoma and malignant dermal analogue tumor according to WHO [2]. BCAC of the salivary gland is an uncommon (1.6%) subtype of all salivary gland neoplasms, accounting for 2.9 percent of all malignant neoplasms [3]. The bulk of cases is seen in the parotid (90%) and submandibular glands, with only a few cases documented in the minor salivary glands and other otorhinolaryngology areas [4,5]. BCAC usually tends to occur in the 6th and 7th decades and there is no gender predilection. Treatment options for BCAC were documented in the literature as surgery, radiotherapy, and chemotherapy [4]. In this study, we discuss an uncommon case of a 60-year-old male with BCAC that originated in the nasopharynx and describe the clinicopathological features of the case. To our knowledge, this case is only the second such case reported to date in the nasopharynx.

## Case presentation

**Figure 1:**
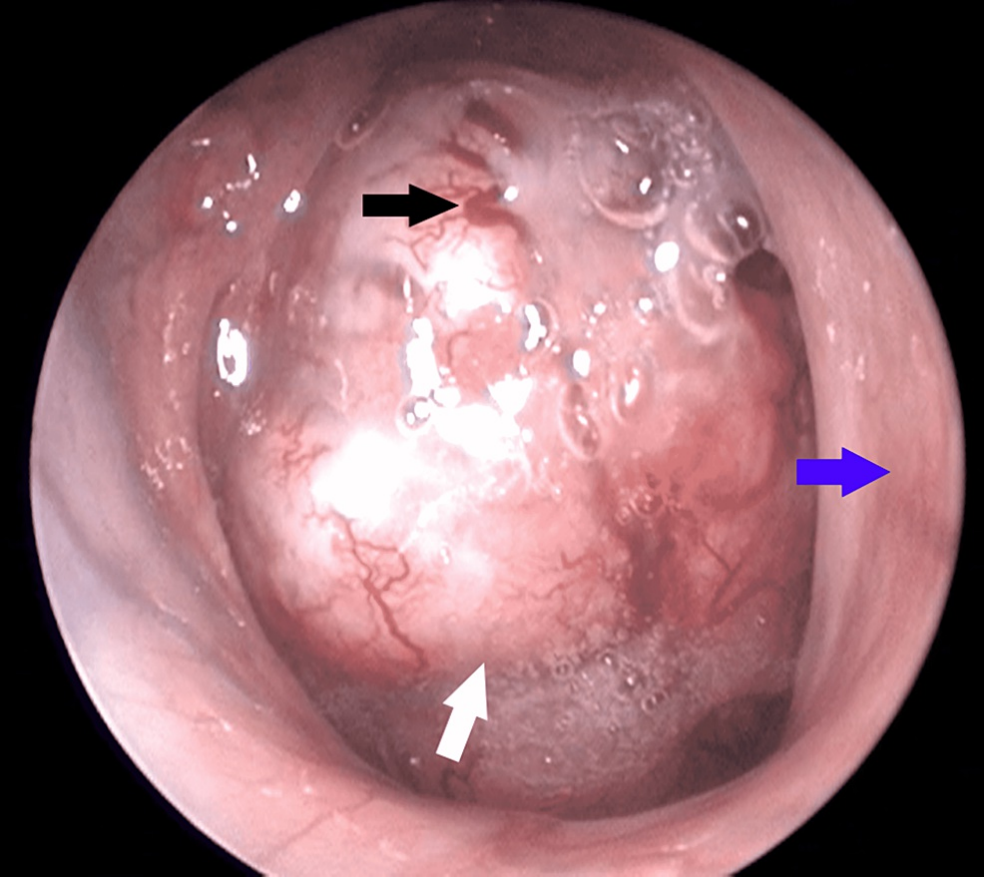
Endoscopic view of the right nasal cavity and nasopharynx at first admission White arrow: Nasopharyngeal mass; Blue arrow: Nasal septum; Black arrow: Areas with vascularization.

A 60-year-old male patient was admitted to the otorhinolaryngology clinic due to nasal congestion, differentiation of tone of voice, and hearing loss in the left ear. He stated that these complaints were present for five months and worsened over time. The patient had no history of headache or epistaxis. The patient had been smoking one pack of cigarettes a day for 40 years. Coronary angiography was performed due to chest pain and a stent was implanted one month before the patient applied to our clinic. Therefore, he was taking bemiparin sodium (1×1, 2500 IU/0.2 ml, subcutaneous), acetylsalicylic acid (1×1, 100 mg, oral), and clopidogrel hydrogen sulfate (1×1, 75 mg, oral). Physical examination revealed a rigid mass originating from the nasopharynx, extending to the inferior of the nasopharynx, with abundant vascularization (Figure 1). 

**Figure 2:**
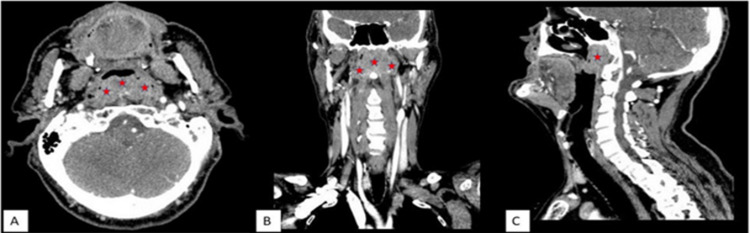
Contrast-enhanced CT images From left to right, axial (A), coronal (B), and sagittal (C) plane images The mass largely obliterates the nasopharyngeal air column, with lobulated contours, heterogeneous contrast (containing non-enhanced areas that may belong to cystic-necrotic areas), causing bilateral mastoid effusion by filling the posterior and both lateral walls of the nasopharynx (bilateral lateral pharyngeal recesses = Rosenmüller fossa) more prominently on the left. The mass had 22x52x36 mm (anterior-posterior x transverse x craniocaudal) dimensions (red asterisks). There were no bony erosion in skull base or enlarged cervical lymph node in the neck.

In addition, the patient had hyponasality, otitis media with effusion in the left ear, and moderate conductive hearing loss was detected in the left ear with pure tone audiometry tests. Examination of cranial nerves and laryngeal functions were normal. There were no palpable cervical lymph nodes on neck examination. Complete blood count and routine biochemical tests were within normal limits. Contrast-enhanced head-neck computed tomography was performed to evaluate the borders of the mass in more detail before biopsy (Figure 2).

**Figure 3:**
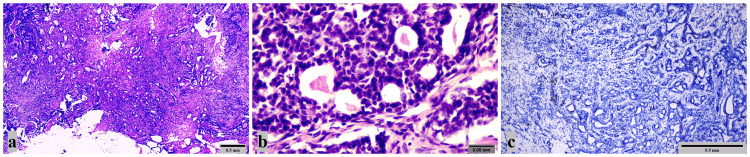
Pathology images of salivary gland adenoma (a): Monotonous epithelial cell groups with round dark nuclei, indistinct nucleoli, cribriform and trabecular structures are seen. There is no mitosis or necrosis (x40; Hematoxylin&Eosin). (b): Monotonous cells with hyperchromatic nuclei and unclear nucleoli are seen (x400; Hematoxylin&Eosin). (c): Positive staining with Ki-67 was observed in 1-2% of cells in tumoral areas (x100; Ki-67).

Punch biopsy was taken from the mass with difficulty because the tumor mass was very strict. The biopsy result was reported as salivary gland adenoma (Figure 3).

**Figure 4:**
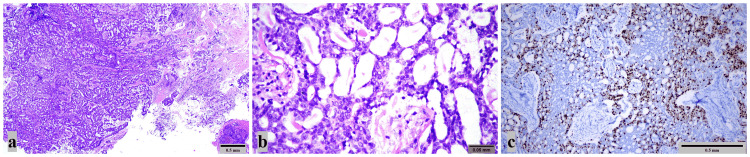
Pathology Images of Basal Cell Adenocarcinoma a: Tumor with extensive cribriform development, forming solid areas, and infiltrating surrounding tissues. Less than 5% necrosis was seen in focal areas (x40; Hematoxylin & Eosin). b: Atypical cells with large vesicular nuclei and prominent nucleoli are seen (x400; Hematoxylin&Eosin). c: Positive staining with Ki-67 was observed in 50% of the tumoral areas (x100; Ki-67).

Due to the presence of a benign nasopharyngeal mass, it was decided to excise the mass completely under general anesthesia. All antiaggregant and anticoagulant treatments used by the patient were stopped and subcutaneous enoxaparin sodium treatment (2×1, 80 mg, s.c.) was initiated instead a week before the surgery. Ventilation tube insertion in the left ear and endoscopic nasopharyngectomy were performed under general anesthesia. It was very difficult to scrape the mass from the nasopharynx walls during surgery, and there was no clear cleavage in the mass. Hyponasality recovered after surgery. Histopathological examination of the nasopharyngectomy material reported as BCAC (Figure 4).

A week after the operation, antiaggregant and anticoagulant treatments for the patient were planned as before the operation. In terms of possible metastasis of malignancy, positron emission tomography was performed on the patient and 18F-fluorodeoxyglucose uptake was detected in the whole body at physiological limits, except the left rossenmuller fossa (SUVmax:4.77). The patient consulted with the Radiation Oncology Department to be evaluated for adjuvant radiotherapy. Radiotherapy was planned by the Radiation Oncology Department for complementary treatment of the patient. In the first year after the end of radiotherapy, we recommended the patient have a regular otolaryngologic examination every three months. The patient is currently under close follow-up. An informed consent form was obtained from the patient for this case report.

## Discussion

BCAC is a rare malignancy of the upper respiratory tract that develops from the minor salivary glands [6]. A review published in 2015 reported cases of BCAC in the palate, buccal mucosa, lower gingiva, tongue, sinuses, nasal cavity, upper lip, floor of the mouth, base of the tongue, and oral cavity. The total of these cases is seventy-three and no BCAC was reported in the nasopharynx in this review [4]. Nasopharyngeal BCAC was first reported in 2018 and the current case is added to the literature as the second case seen in the nasopharynx [7]. Therefore, unfortunately, it is not possible to obtain clear information about the incidence, gender predominance, or clinical features of BCAC in the nasopharynx.

Basal cell adenoma, adenoid cystic carcinoma, and basaloid squamous cell carcinoma are all other possible diagnoses for minor salivary gland tumors [6]. Among them, differentiating between BCAC and basal cell adenoma especially is extremely difficult. BCACs differ from adenomas in that they have infiltrative traits, as well as perineural and angiolymphatic invasion, and they may have higher mitotic activity and necrosis [2]. In the BCAC case we present, there was no perineural and perivascular invasion, but it was markedly infiltrative and involved necrosis. In addition, benign tumors almost always have a Ki-67 index <5%. A Ki-67 index of >10% is practically restricted to malignant tumors [8]. The present case had a Ki-67 index of 50% in nasopharyngectomy material whereas punch biopsy had a 1-2%.

Clinical-endoscopic examinations and imaging methods cannot distinguish between the pathological subtypes of adenocarcinoma. Therefore, our most important tool for deciding the histological subtype is a biopsy. A punch biopsy is a very common method for diagnosing nasopharyngeal masses. The advantages of punch biopsy are that it can be performed under local anesthesia and the patient can be discharged on the same day. However, as in our case, situations that mislead the pathologist may occur. We emphasize the need to be more careful especially for tumors of non-epithelial origin in the nasopharynx (e.g. salivary gland tumors). In this case, nasopharyngectomy was performed because the punch biopsy result of the patient was reported as a benign tumor. But the results for the nasopharyngectomy material changed this diagnosis to BCAC. We think that some points should be considered here: 1. the surface of the mass was not cauliflower-like (had a smooth surface), 2. there is clearly visible vascularization on the surface of the mass, and 3. the tissue taken during punch biopsy was not easily separated from the mass. In these conditions, it should be assumed that there is a different tumor other than nasopharyngeal carcinoma (keratinizing, non-keratinizing, and undifferentiated subtypes), which is the most common tumor type in the nasopharynx. In such cases, we believe that taking biopsies, including larger and non-tumoral tissues (healthy tissue of the nasopharynx), would be more beneficial to pathologists for an accurate diagnosis.

Although surgery is the main treatment modality for the treatment of BCACs in the upper respiratory tract other than the nasopharynx, radiotherapy and/or chemotherapy can be added after surgery [4]. Due to its anatomical location, the nasopharynx is an area where tumor surgery is difficult and negative surgical margins are difficult to obtain [9]. Therefore, the radiotherapy option comes to the forefront for nasopharyngeal tumors. The previously-reported nasopharyngeal BCAC was treated with radiotherapy and no recurrence was observed during the 72-month follow-up [7]. In this case, an endoscopic nasopharyngectomy was performed because the first pathology report was a benign tumor. However, radiotherapy was planned to the patient as a complementary treatment because the results for the nasopharyngectomy material reported BCAC and the presence of tumors in the surgical margins.

Minor salivary gland malignancies have a poor prognosis, according to a conventional rule in head and neck oncology. In addition, there are differences in prognosis according to different subtypes of the same tumor. In a study that analyzed the overall survival of sub-histological types of nasopharyngeal adenocarcinomas, the tumor subtype with the longest overall survival was reported to be papillary adenocarcinoma. The median overall survival for papillary carcinoma is 226.9 months. The median overall survival reported for all nasopharyngeal adenocarcinomas in the same study was 60.6 months [10]. Since the histologic subtype of the patient we presented was BCAC, our estimate based on the above-mentioned study data is that the overall survival will be shorter than for the papillary histologic subtype.

## Conclusions

This case report is the second presentation of BCAC originating from minor salivary glands in the nasopharynx. In the differential diagnosis of nasopharyngeal masses, it should be remembered that BCAC may be present. Obvious features of tumoral tissue during endoscopic examination and punch biopsy may be indicative of BCAC. Endoscopic nasopharyngectomy can be performed to remove the tumor during treatment, but because of the anatomical location in the nasopharynx and the difficulty of obtaining a negative margin in nasopharyngeal surgery, radiotherapy can be attempted as the first treatment option or is another reasonable option that can be performed in addition to surgery.
